# Insight into Improved Thermostability of Cold-Adapted Staphylococcal Lipase by Glycine to Cysteine Mutation

**DOI:** 10.3390/molecules24173169

**Published:** 2019-08-30

**Authors:** Jiivittha Veno, Raja Noor Zaliha Raja Abd Rahman, Malihe Masomian, Mohd Shukuri Mohamad Ali, Nor Hafizah Ahmad Kamarudin

**Affiliations:** 1 Enzyme and Microbial Technology Research Centre, Universiti Putra Malaysia, Serdang 43400, Selangor, Malaysia; 2 Department of Microbiology, Faculty of Biotechnology and Biomolecular Sciences, Universiti Putra Malaysia, Serdang 43400, Selangor, Malaysia; 3 Centre of Vaccine Research, School of Science and Technology, Sunway University, Bandar Sunway, Selangor 47500, Malaysia; 4 Department of Biochemistry, Faculty of Biotechnology and Biomolecular Sciences, Universiti Putra Malaysia, Serdang 43400, Selangor, Malaysia; 5 Centre of Foundation Studies for Agricultural Science, Universiti Putra Malaysia, Serdang 43400, Selangor, Malaysia

**Keywords:** staphylococcal lipase, cold-adapted, thermostability, *in silico* modeling and analysis

## Abstract

Thermostability remains one of the most desirable traits in many lipases. Numerous studies have revealed promising strategies to improve thermostability and random mutagenesis often leads to unexpected yet interesting findings in engineering stability. Previously, the thermostability of C-terminal truncated cold-adapted lipase from *Staphylococcus epidermidis* AT2 (rT-M386) was markedly enhanced by directed evolution. The newly evolved mutant, G210C, demonstrated an optimal temperature shift from 25 to 45 °C and stability up to 50 °C. Interestingly, a cysteine residue was randomly introduced on the loop connecting the two lids and accounted for the only cysteine found in the lipase. We further investigated the structural and mechanistic insights that could possibly cause the significant temperature shift. Both rT-M386 and G210C were modeled and simulated at 25 °C and 50 °C. The results clearly portrayed the effect of cysteine substitution primarily on the lid stability. Comparative molecular dynamics simulation analysis revealed that G210C exhibited greater stability than the wild-type at high temperature simulation. The compactness of the G210C lipase structure increased at 50 °C and resulted in enhanced rigidity hence stability. This observation is supported by the improved and stronger non-covalent interactions formed in the protein structure. Our findings suggest that the introduction of a single cysteine residue at the lid region of cold-adapted lipase may result in unexpected increased in thermostability, thus this approach could serve as one of the thermostabilization strategies in engineering lipase stability.

## 1. Introduction

Stability plays a potent role in determining the level of protein functionality. Higher stability makes a protein more economical as it reduces enzyme turnover and becomes more robust in function, such as in extreme conditions. The term ‘stability’ denotes a protein resistance towards various deleterious factors such as heat or denaturants that could affect its molecular integrity or biological function upon exposure [1]. Many native proteins or enzymes have been studied for their unique intrinsic properties, for instances, thermostable enzymes from thermophiles, cold-active enzymes from psychrophiles and organic solvent stable enzymes from organic solvent tolerant microbes. The ability of native enzymes to demonstrate such stability has prompted the search for the underlying factors at molecular and structural levels.

Structural adaptation strategies of the extremophiles are unique. Variations in the sequence and structure of psychrophilic enzymes to their higher counterparts (mesophilic and thermophilic enzymes) have significantly revealed the influence of specific trends of amino acids to their adaptation strategies. In cold-adapted enzymes including lipases, increased global flexibility or local flexibility serves as the most accepted notion for cold-adaptation although local flexibility which is more important lacks experimental information [2,3,4]. The adaptation strategies of each cold enzyme, however is comparatively different and unique among them. *Candida antarctica* lipase (CAL) is by far the most widely studied cold active lipase isolated from the Antarctic. The CAL-A and CAL-B lipases were identified and both exhibited distinct physicochemical characteristics. In CAL-B, the flexible active site is suggested to be important for its high catalytic activity at low temperatures [5].

As enzymes particularly, lipases are an interesting subject to various biotechnological industries, finding a catalyst that suits the industrial demands is often challenging. Despite the auspicious qualities found in native lipases, insufficient protein stability in reaction media has restricted their employment in various industrial processes. Since many industrial applications operate at temperatures above 45 °C, the use of biocatalyst can be problematic as high temperatures will unfold the protein. Alternatively, protein engineering approaches have been widely used to alter the protein scaffold to increase protein stability including thermal resistance.

Directed evolution is one of the powerful methods to modify enzymes without prior knowledge of the three-dimensional protein structure. This method has been successfully used to generate thermally stabilized mutant proteins [6]. However, it is important to note that methods of stabilizing proteins have shown a trade-off between rigidity needed for stability and flexibility required for activity in most enzymes [7]. Hence, it is critical to aim at maintaining the enzymatic activity while improving the stability. Designing novel enzymes that sustain both activity and stability at temperatures which vary from their physiological temperatures would permit the most effective use of enzymes in a broad range of biotechnological reactions in many areas such as biopharmaceuticals, agro-food, and environment [8].

Over the years, many studies have been conducted to investigate the intrinsic stability of thermophilic proteins that render them to thermo-stabilization. The most commonly predicted factors are associated with their primary structure, hydrophobic interactions among the hydrophobic residues, salt bridges due to the presence of charged residues, hydrogen bonds between surrounding water molecules and the surface exposed hydrophilic residues, packing efficiency and amino acid replacement in the interior and exterior of the secondary structures, decreased occurrence of thermolabile residues loop stabilization and defense against covalent destruction [9,10,11].

Previously, mutant G210C from *S. epidermidis* AT2 lipase was laboratory evolved and demonstrated a significant improvement in its thermostability profile [12]. The cold-adapted enzyme, which is initially active at 25 °C shows about 85% stability at 50 °C (while retaining its activity at 25 °C) after a random introduction of a cysteine at residue 210 in replacement of glycine. It is worth mentioning that this cysteine represents the only cysteine residue found in the amino acid composition. Interestingly, this residue is also absent in the mature sequence of most staphylococcal lipases. In this work, we sought to trace the dominant factors that led to the altered thermal stability profile of this cold-adapted lipase by a single point mutation through comparative molecular dynamics (MD) simulation at two temperatures.

## 2. Results

### 2.1. Structure Prediction and Model Validation of rT-M386 and G210C Lipases

In earlier studies, the C-terminal truncated lipase from *S. epidemidis* AT2 (rT-M386) was mutated using error-prone PCR. From the resultant mutant library, one mutant with a single point mutation (G210C) showed more than 85% (401 U/mg) stability at 50 °C, shifting the temperature profile of this cold-adapted lipase [12]. 

To assess the effects of mutation on structural conformation of both wild-type and mutant G210C, we conducted in silico modeling and MD simulation. The PSI-BLAST and SWISS-MODEL template search showed that out of the top template homologs, crystal structure of *Staphylococcus hyicus* lipase (PDB ID: 2HIH) had the highest match to the target sequences with 51% identity with 1% gap between the two sequences (Figure S1). To the best of our knowledge, 2HIH represents the only 3D crystal structure available for staphylococcal lipase family (Family I.6) in Protein Data Bank (PDB). Therefore, it serves as the most reliable template compared to other lipases from other families such as *Bacillus* with less than 40% identity.

The two models were subjected to validation. Stereochemical quality and overall model qualities of rT-M386 and G210C lipase models were evaluated using PROCHECK and ERRAT. The Ramachandran plot of rT-M386 predicted structure showed that the percentage of residue existed in the most favored region is 89.3%, 10.1% in the additionally allowed region, 0.6% resided in generously allowed region and none in the disallowed region (Figure S2a). For G210C, more than 90% of the residues were in most favored region, 8.8% in the additionally allowed region residues, 0.6% in the generously allowed region and none in the disallowed region (Figure S2b). ERRAT2 analysis showed that both models exhibited an overall quality factor of 97.3% and 95.6% at below 95% rejection limit, respectively (data not shown) suggesting that the two models are acceptable and reliable for further structural and MD analysis.

**Figure 1:**
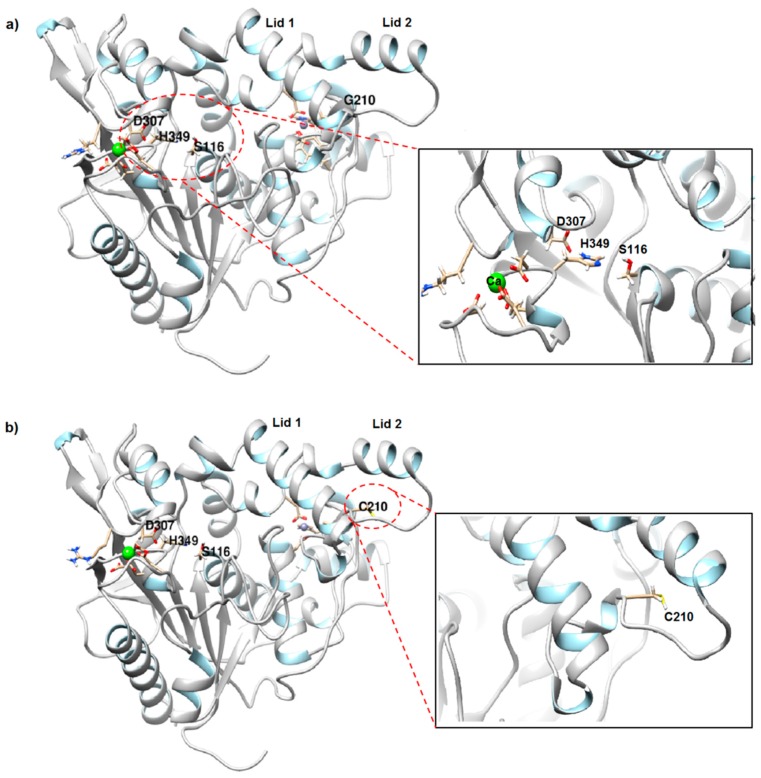
Predicted 3D structure of lipases. (**a**) rT-M386 and (**b**) mutant G210C. Ca^2+^ and Zn^2+^ are colored in green and gray solid circle, respectively. Catalytic triad of rT-M386 and G210C comprised of S116, D307 and H349 situated on β-sheet, loop and turn, respectively. Mutation point is labeled as C210. Models are generated by Chimera in linear ribbon.

Both predicted rT-M386 and G210C lipases adopted the typical α/β hydrolase fold formed in the core region and the β- sheets are surrounded by α-helices (Figure 1). Three catalytic residues in both lipases; Ser116, Asp307 and His349 are placed at a highly conserved geometry throughout lipase families; a turn following strand 5, loop 9–13, and loop 12–8, respectively. The active serine, Ser116 appears at the pentapeptide of G-X_1_-S-X_2_-G motifs, where X_1_ and X_2_ are denoted by His115 and Met117, respectively. 

rT-M386 and G210C revealed two metal binding sites, Ca^2+^ and Zn^2+^ (Figure S3). Ca^2+^ ion is held by five aspartate residues (Asp 283, Asp 348, Asp 351, Asp 356 and Asp 359) in a pentahedral coordination of while Zn^2+^ binding site is coordinated in pentahedral and tetrahredral configuration, respectively. Zn^2+^ was believed to play an important role in lid regulation and found conserved in staphylococcal lipases that do not display stability at elevated temperature.

### 2.2. Comparative Structural Analysis of rT-M386 and G210C Lipase Models

#### 2.2.1. Superimposition of rT-M386 and G210C

Overall folding of both lipases is almost similar and superimposition of backbone co-ordinates of wild-type and mutant showed Root Mean Square Deviation (RMSD) of 1.58 Å over 5978 matched atoms. According to Carugo and Pongor (2002) [13], RMSD value defines the degree of differences in the superimposed structures at which identical structures will exhibit zero value of RMSD. In general, we observed no apparent change in G210C structure compared to the wild-type.

The mutation point was located on the loop connecting the two lids (lid 1 and lid 2) covering the active site (Figure 1). As observed in both structures, Gly210 (in wild-type) and Cys210 (in mutant) are located at distances between 25.9 Å to 26.6 Å from the active site serine and will not likely to perturb neither the orientation of the triad nor directly the enzymatic activity.

The metal binding sites (Ca^2+^ and Zn^2+^) appear to be somewhat different between G210C and its wild-type. The distance between Ca^2+^ and the adjacent Ca^2+^ binding residues of G210C lipase is slightly closer (between 1.92–2.29 Å), as compared to rT-M386 (between 2.24–2.46 Å) suggesting a stronger contact of Ca^2+^ with five Asp residues present in G210C. On the other hand, the configuration of Zn^2+^ binding site fairly differs in both structures. The tetrahedral configuration observed in G210C is similar to that found in many thermostable lipases and it has been reported to be conserved among thermostable lipases [14,15]. The same coordination was reported in mesostable *S. hyicus* lipase [16]. In the case of G210C, the Zn^2+^ is held by two aspartates (Asp64, Asp236) and two histidines (His84, His90). Tyr80 which is part of the Zn^2+^ binding ligands in the wild-type was missing in mutant G210C. 

#### 2.2.2. Cysteine Substitution

Generally, free cysteines have been reported to participate in a variety of roles in protein structure and function, including disulfide bond, dimerization, hydrogen bond, metal coordination, enzyme catalysis, redox regulation and thermal stability [17]. Cysteine has also been known to behave as a strong hydrophobic amino acid that stabilizes the globular protein compared to serine, a polar amino acid and glycine, a hydrophobic and non-polar amino acid. The hydrophobicity of free cysteine is suggested due to its prevalence in the hydrophobic cluster, along with residues such as Met, Trp and Tyr along with the fact that SH group does not react with water molecule [18]. Addition of Cys210 between two other existing hydrophobic amino acids Trp209 and Phe211 in replace of glycine attributed to the increased of hydrophobic effect in the loop stretch. In contrast, the reduced hydrophobic effect observed in the wild-type where glycine is present could have caused the water molecules to penetrate into the protein, resulted in swelling and denaturation and subsequently alter the tertiary conformation of the native state [19]. Moreover, glycine is known as secondary structure breaker due to its inability to protect the backbone of the hydrogen bond with extremely small side chain and this will disrupt the secondary structure of a protein [20]. 

In a study performed by Qiu and co-workers (2015) [21], conservative mutation of Cys to Ser caused a slight loss of thermostability and location of the free cysteine (whether buried or exposed) was a contributing factor to the stabilizing and destabilizing effect. According to NetSurfP-2.0 prediction, the relative surface accessibility (RSA) of amino acid of below 25% is considered buried while above 25% is exposed [22]. Based on NetSurfP-2.0 results (Figure S4), Gly210 is exposed to the surface with an RSA value of 30%. When mutated to cysteine, this amino acid became buried and the RSA value dropped to 11%. The tight packing of cysteine due to its bulky side chain in mutant G210C is likely to impart stabilization to the loop structure. 

#### 2.2.3. Hydrogen Bond and Salt Bridge Analysis

Further analysis showed that substitution of glycine to cysteine resulted in formation of additional hydrogen bond (Table 1). It is well accepted that hydrogen bond is one of the important factors that contribute to thermostablity. Based on Yet Another Scientific Artificial Reality Application (YASARA) analysis, rT-M386 displayed a total of 334 hydrogen bonds while mutant G210C exhibited additional 15 hydrogen bonds making a total of 349. This contributes to an extra 235.8 kJ/mol in G210C and could be important to the increased stability, which in turn correlated with temperature shift. 

**Figure 2:**
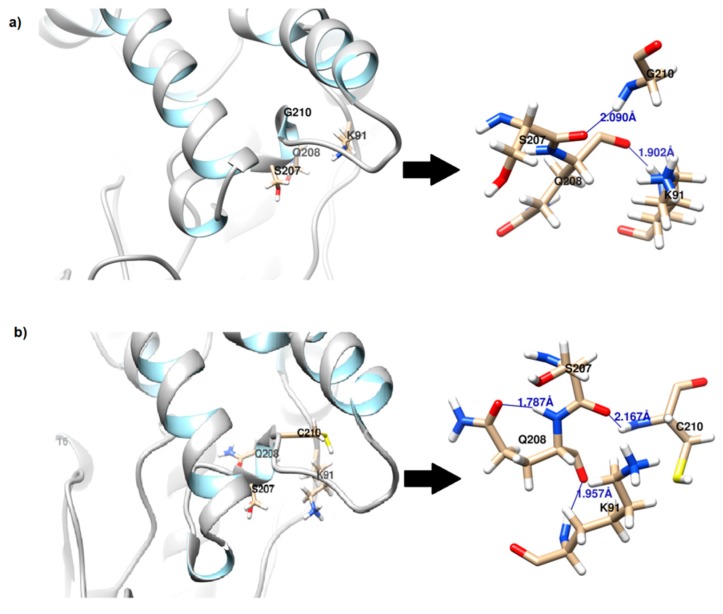
Local hydrogen bond between residue 210 and its neighboring residues as observed in (**a**) rT-M386 and (**b**) mutant G210C. An additional intraresidue hydrogen bond is formed at Q208 in mutant G210C with a distance of 1.787 Å.

The local change however is small where the hydrogen bond that forms between the N-atom of Gly210 to O-Ser207 at a distance of 2.09 Å is replaced with Cys210/N-Ser207/O with a distance of 2.17 Å and binding energy of 13.37 kJ/mol (Figure 2). No additional hydrogen bond was detected between Cys210 and its neighboring residues. Interestingly, Q208 which lies in the same vicinity forms an intra-residue H bond with a distance of 1.79 Å (Figure 2b), adding an extra hydrogen bond to the loop. 

**Figure 3:**
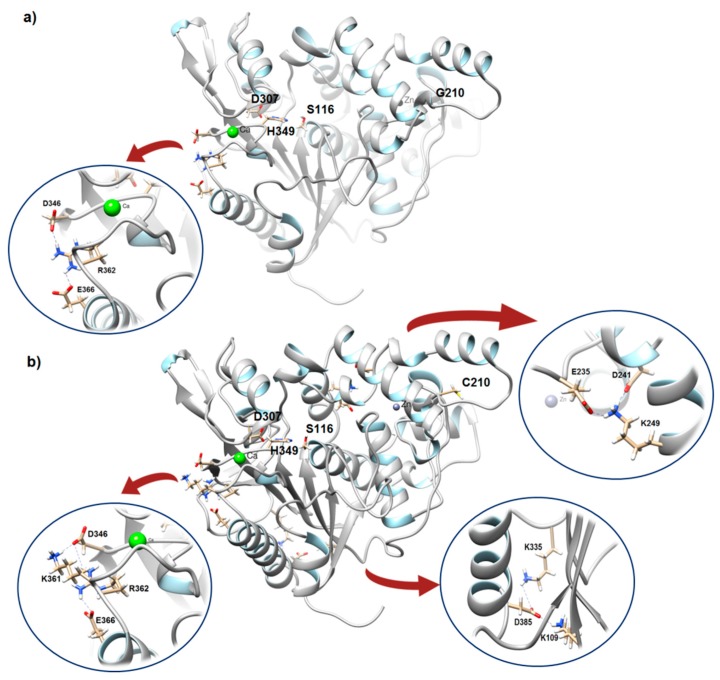
Salt bridge/Ion pair network of rT-M386 and mutant G210C. The catalytic triad residues are labeled as S116, D307 and H349. The salt bridge networks are represented in circle. Contacts formed between residues (**a**) in rT-M386: R362-K361/R263-D346 and (**b**) in G210C: R362-E366/R362-D346, K109-D385/K335-D385 and K249-E325/ K249-D241.

Electrostatic interaction or salt bridge is among the first dominating notions in protein stabilization and thermostable proteins established in a higher frequency occurrence of salt bridges compared to their mesophilic counterparts. To predict the formation of salt bridges between the ion-pairing residues within a 4 Å distance in both model structures, we used Evaluating the Salt BRIdges in Proteins (ESBRI) software. In total, both wild-type and mutant consist of 45 negatively charged residue (Asp (D) + Glu (E)) and 42 positively charged residues (Arg (R) + Lys (K)). Mutant G210C displayed a higher number of salt bridges compared to the wild-type. One salt bridge network made up of three different amino acid residues was seen in the wild-type (Figure 3a). In contrast, three salt bridge networks made up of thirteen different amino acid residues were identified in mutant G210C; one is located in close proximity to the catalytic triad of G210C and Ca^2+^ binding site whereas the other two are found near to the lid and C-terminal region (Figure 3b). 

The salt bridges between K361-D346 and R362-E366/R362-D346 in mutant G210C are at the helix-coil motif holding the Ca^2+^ ion and very close to the active site residues especially D307 and H349. The contact between K361-D346 was not found in the wild-type suggesting a larger salt bridge network was formed in the same vicinity. We further identified whether the salt bridges are buried or exposed. An exposed salt bridge is suggested to have one residue with RSA value >35% and another with >25% [23]. According to the RSA value calculated by NetSurfP-2.0, three residues exhibited RSA value of more than 35% while R362 with 32% indicating that the two salt bridges are in exposed state. Meanwhile, another two salt bridge networks between residues K109-D385/K335-D385 and K249-E325/ K249-D241 are exposed and slightly buried, respectively. K109-D385/K335-D385 are at the strand-coil motif while the latter are found at helix-coil motif. The pairing to more than one residue may hinder the inherent flexibility of the coil and is likely to promote a stronger stabilizing effect that holds different secondary elements of the enzyme. 

#### 2.2.4. Cation-π Interactions 

Contribution of cation-π interaction in protein stability has been increasingly recognized as important apart from hydrogen bond, hydrophobic interaction, and salt bridges. The cationic side chain of charged residues namely arginine and lysine would experience a cation-π interaction when it is closed to aromatic side chain (Trp, Tyr and Phe) due to geometry bias [24]. The strength of cation-π is generally associated with increased temperature. At 25 °C, the interaction is said to be weakly stabilizing [25]. To evaluate the influence of this factor to the thermostability of mutant G210C, the number of cation-π interaction was calculated. Interestingly, we observed some differences in cation-π interactions in mutant G210C and its wild-type. Lys6 and Lys193 interacted with Tyr49 and Tyr219/Tyr222, respectively in G210C. Meanwhile, in rT-M386, Arg95 forms cation-π interaction with Tyr97 whereas Arg190 contributed four interactions with Trp232. Our results account for the prevalence of Arg in the interaction though Lys is a more common residue in rT-M386. One explanation could be the larger side chain of arginine permits a stronger interaction and likely to benefit in higher polarity environment [26,27]. Close to the mutation site, Lys193 at lid 1 participates in the formation of cation-π interaction by making a contact with two tyrosines (Lys193-Tyr219 and Lys193-222) while in rT-M386, only one interaction was found with Tyr49. The double dentate mode is presumed to play essential role in stabilizing the protein especially the lid region. Furthermore, Tyr265 in the new mutant makes a stronger interaction with Arg341 via seven interactions while only four interactions were observed in the wild-type at the same contact residue. 

### 2.3. MD Simulation of rT-M386 and G210C Lipases 

**Figure 4:**
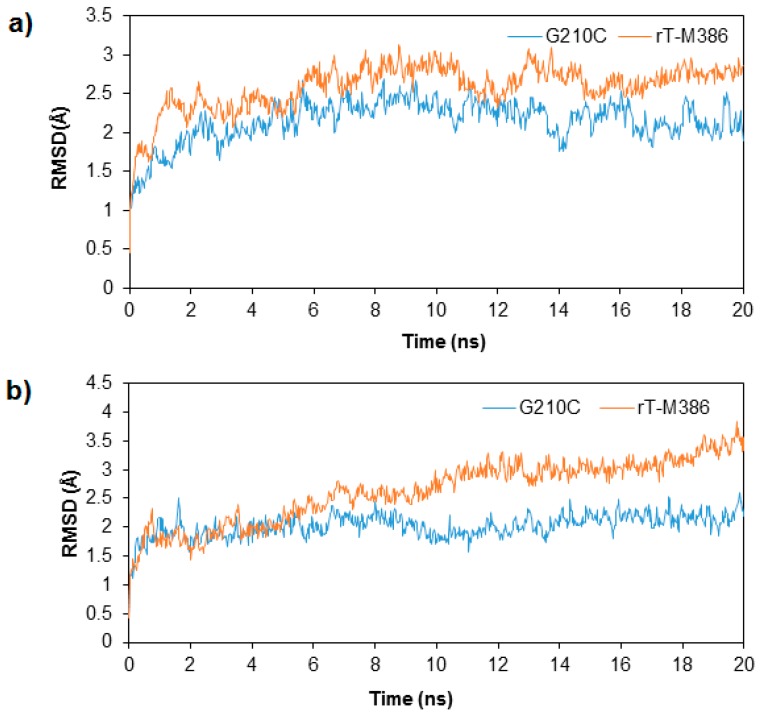
Root Mean Square Deviation (RMSD) profile for rT-M386 and G210C as per time. (**a**) at 25 °C (**b**) at 50 °C. The simulation was analyzed over 20 ns trajectories. The RMSD of rT-M386 and G210C lipases are indicated by red line and blue line, respectively.

To investigate the influence of temperature to the dynamics of the enzyme, MD simulations were conducted and analyzed by means of RMSD, Root Mean Square Fluctuation (RMSF), Radius of Gyration (Rg) and solvent accessible surface area (SASA) values. The RMSD values which were computed for 20 ns trajectories on Cα atoms for rT-M386 and G210C lipases was analyzed at 25 and 50 °C (Figure 4). In the beginning of trajectory (t = 0 ns) at 25 and 50 °C, RMSD displayed a value of about 0.4 Å for both lipases. This signified the movement in Cα backbone of both lipase structures during thermalization and equilibration periods. During the first 0.7 ns of the simulation at 25 °C, RMSD value of rT-M386 and G210C lipases increased rapidly to 1.6 Å but G210C lipase structure stabilizes at a range of 1.6 to 2.5 Å until the end of simulation (t = 20 ns). At 9 ns, rT-M386 reached the highest RMSD value of 3.0 Å, while the RMSD of G210C was only 2.5 Å at the same trajectory. The G210C lipase maintained the stability of the structure during the entire 20 ns. Moreover, the rT-M386 lipase structure was less stable at 50 °C as the RMSD value dramatically increased up to 3.8 Å starting from 5 ns compared to G210C where its highest value was only 2.6 Å throughout 20 ns trajectories. In the experimental work reported earlier, the half-life of rT-M386 lipase at 50 °C was 10 min while the G210C lipase was stable at 50 °C with a half-life of 2.5 h [12]. Thus, these computational results positively correlated with the experimental work, suggesting that the mutant is relatively more stable and demonstrated high catalytic performance at elevated temperature. 

To examine the flexibility of residues, we measured the local dynamic change around the mutated site, defined as RMSF of Cα backbone against each residue. The RMSF curve showed similar pattern to the RMSD of Cα backbone per residue previously reported [12]. As both lipases exhibited lipolytic activity at 25 °C, it was presumed that the conformational flexibility would not be substantially different when simulated at low temperature. Our previous experimental work showed that although the mutant demonstrated improved stability at high temperature, the enzyme was still active at 25 °C. In agreement, at 25 °C simulation, no obvious change in the RMSF curves between rT-M386 and G210C was observed. The largest amplitude of fluctuation occurred in regions involved in catalysis; notably the lid region apart from terminal ends and several loops (data not shown). In CAL-B lipase, flexibility and hydrophobicity of the active site have enabled the lid to exhibit optimal dynamics at cold temperature [5]. Likewise, the presence of highly mobile lid in rT-M386 explains its functionality at low temperature however higher temperature may lead to unfolding of the overall conformation of rT-M386 due to large local movement. At 50 °C, significant alterations in structural scaffolds were recorded especially at the loop-lid 2 motif where the mutation lies. For mutant G210C, the rise in temperature does not only seem to cause lower fluctuation in RMSF, but also increases in the number of residues that become more rigid in position. 

**Figure 5:**
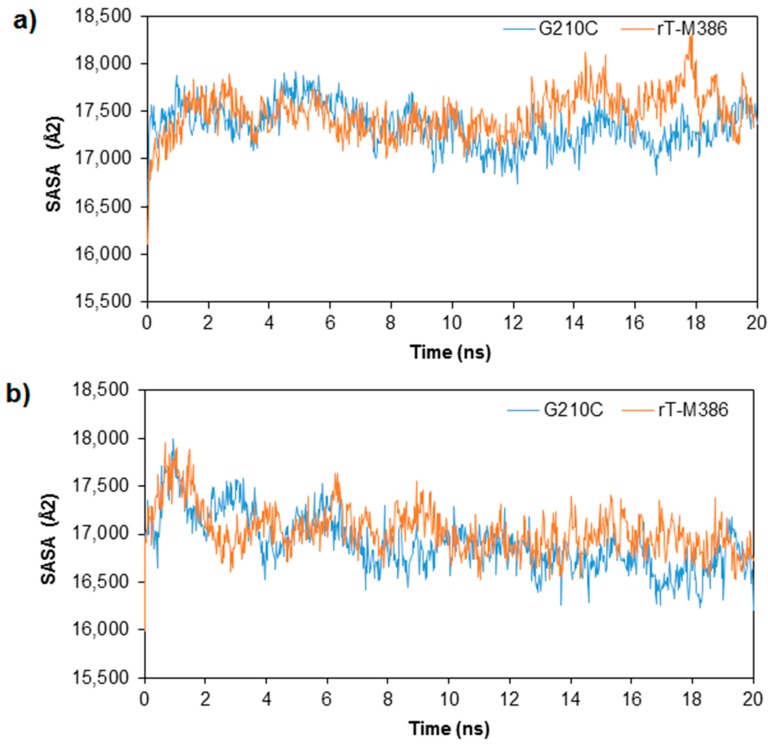
Solvent accessible surface area (SASA) of rT-M386 and G210C as per time. (**a**) at 25 °C (**b**) at 50 °C. The simulation was analyzed over 20 ns trajectories. The SASA of rT-M386 and G210C lipases are indicated by red and blue lines, respectively.

Solvent accessible surface area (SASA) of rT-M386 and G210C lipases was computed using an analytical algorithm for 20 ns trajectories in the water system at 25 and 50 °C (Figure 5). In general, almost a similar pattern of SASA values (16,100–17,800 Å^2^) were recorded for both lipases during the course of simulation. However, a moderate decreased of SASA to a value of 16,207 Å^2^ was observed at the end of high temperature simulation for G210C suggesting that the folding process in the mutant lipase structure has occurred more proficiently than in rT-M386 structure with 16,723 Å^2^. The changes in temperatures did not greatly affect the SASA of both lipase structures. One explanation could be the accessible surface encloses a volume (Å^3^) of each lipase are comparable. The rT-M386 and G210C lipases covered about 70,567.25 and 69,984.68 Å^3^, respectively (values are generated after superimposition by YASARA). 

**Figure 6:**
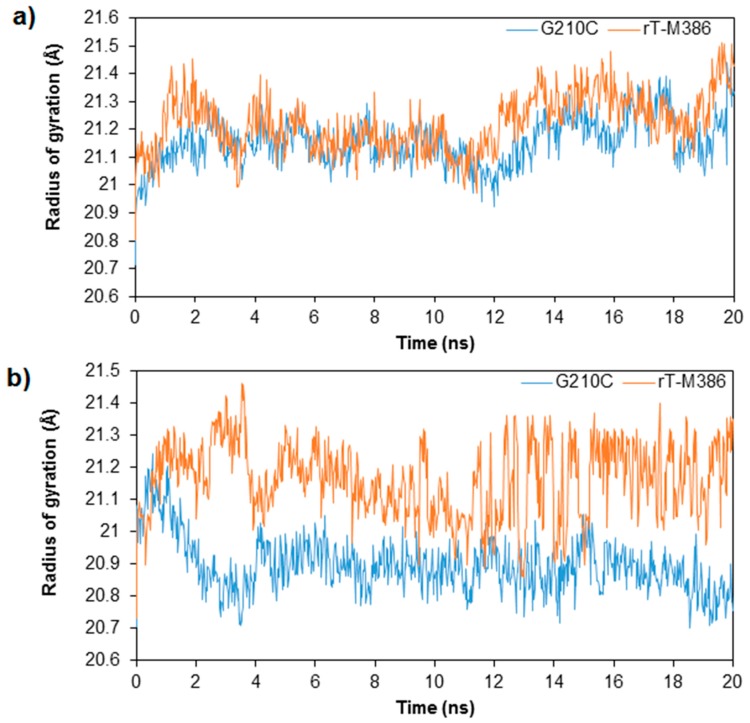
Radius of Gyration of the rT-M386 and G210C lipases as per time. (**a**) at 25 °C (**b**) at 50 °C. The simulation was analyzed over 20 ns trajectories. The radius of gyration of rT-M386 and G210C lipases are indicated by red and blue lines, respectively.

In terms of structural compactness of the two lipases at two different temperatures, the Rg plot demonstrated quite an important difference between rT-M386 to G210C at low and high temperature simulations (Figure 6). At both temperatures, the Rg values for rT-M386 were higher compared to its mutant. At 25 °C, the radius of rT-M386 expanded to a range of 21.4 Å whereas the Rg value of G210C was 20.7 Å. Meanwhile at 50 °C, the Rg values for G210C decreased to 20.7 from 21.3 Å. Reduction in Rg value depicted that G210C is structurally more compact than its wild-type. 

### 2.4. Structural Dynamics of Mutant G210C and Its Wild-Type

**Figure 7:**
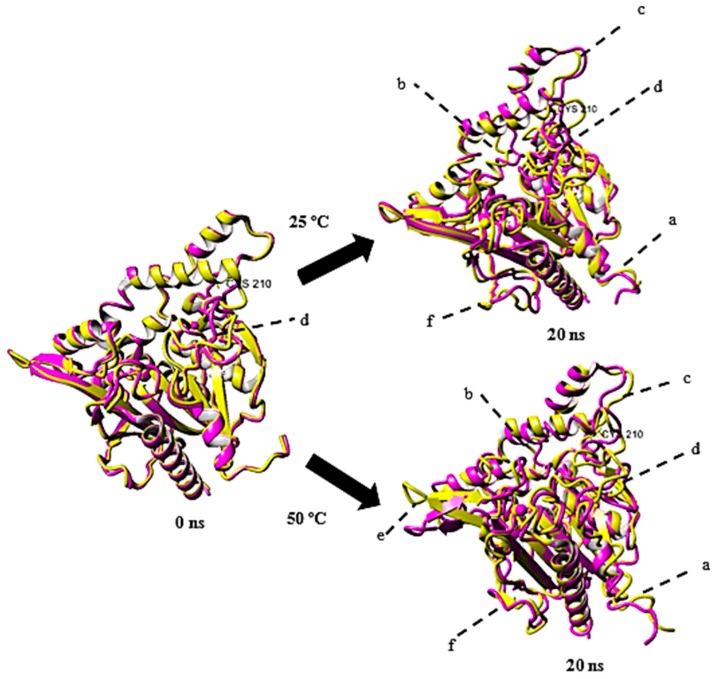
Superimposition of rT-M386 and G210C lipase predicted structures. The structure of rT-M386 and G210C lipases are colored in magenta and yellow, respectively. (**a**) residue 1–30 (**b**) residue 136–143 (**c**) residue 173–217 (**d**) residue 230–235 (**e**) residues 273–277 (**f**) residue 324–326. Mutation point is labelled as CYS 210. Figure is generated using YASARA.

Snapshots of the structural conformation of rT-M386 and G210C lipases were extracted at 20 ns and superimposed to compare their structures based on whole atoms at 25 and 50 °C (Figure 7). The structural conformation of both lipases before simulation (*t* = 0 ns) were used as a control. Superimposition at 0 ns resulted in RMSD of 1.6 Å over 5982 matched atoms and increased to 3.2 Å after 20 ns simulation at 25 °C. Whereas, a higher RMSD (3.5 Å over 5982 matched atoms) of the superimposition was recorded at 50 °C. It is obvious that the high temperature increased the movement of Cα backbone resulting in larger deviation and fluctuations from the initial structure (Figure 7).

At 0 ns, major conformational changes were observed only at region labeled as d. As the simulation continued, larger amplitude of movement was recorded at the N-terminal of the lipases (residue 1–30), along the loop (residue 136–143), lid region (residue 173–217), near α-helices (residue 230–235), near β-sheet (residues 273–277) and near C-terminal (residue 324–326) at both 25 and 50 °C. rT-M386, in general, displayed higher RMSF values than G210C at most of the regions. Also, a β-strand structure near the C-terminal region (labeled as f) of the wild-type lost its conformation and turned into a coil structure after 20 ns simulation. 

**Figure 8:**
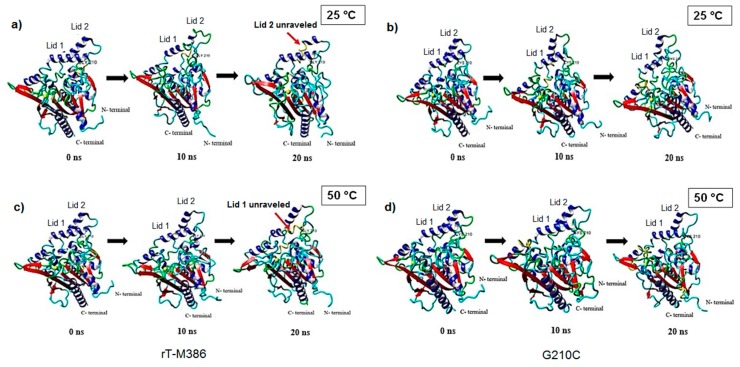
The snapshots of rT-M386 and G210C models at 0, 10 ns and 20 ns simulations. (**a**) rT-M386 at 25 °C; (**b**) G210C at 25 °C; (**c**) rT-M386 at 50 °C; and (**d**) G210C at 50 °C. The α-helices in the lid region of rT-M386 started to unravel at both temperatures towards the end of simulations. The α-helices, β-strands, 3_10_-helix, turn and loops are shown in blue, red, yellow, green, and cyan colors, respectively. Residues of Gly/Cys 210, lids and N- and C- termini are labeled, accordingly. Models are generated by YASARA in linear ribbon.

The conformational changes that occurred in the structure of rT-M386 and G210C lipases at respective temperature; between 0, 10 and 20 ns trajectory are shown in Figure 8. We selected the three trajectories as they portrayed the obvious structural differences during the course of simulations. As seen in Figure 8a, lid 2 (residues 218–229) of the wild type which is located close to the mutation point began to unravel and lost its secondary structure at 25 °C while at 50 °C, lid 1 was unfolded to a longer 3_10_-helix (Figure 8c). In contrast, such observations were not seen in the new mutant. The lid region of G210C lipase were well maintained at both 25 and 50 °C as shown in Figure 8b,d, respectively.

## 3. Discussion

Enhancement of protein thermostability has been driven by many factors and stabilization of lid domain is one of the factors defining the trait of thermostable lipases [28]. Secundo et al. (2006) [29] reported that the lid greatly influences the activity, specificity and conformational stability of the lipases. The flexibility of the lid especially in lipases enables the enzyme to change its conformation from close to open upon interaction with substrates apart from the hydrophobicity required for interfacial activation. Since Cys210 is located on the surface exposed loop in close proximity with the lid helices, special emphasis was given to the lid to observe any plausible change that could cause the thermostability shift from 25 to 45 °C and a longer half-life at 50 °C (2.5 h) compared to its wild type (10 min) [12]. 

The well-maintained lid region in mutant G210C at both low and high temperature simulations; 25 and 50 °C suggested that substitution of glycine to cysteine at the loop connecting lid 1 lid 2 could be an important stabilizing factor that accounts for its stability at elevated temperatures especially at 40–50 °C. This helix-loop-helix motif allows the protein chain to fold back on itself and produce a compact shape which provides stability to the lid. The free cysteine is presumed to have substantial role in stabilizing the two α-helices. Since free cysteine is reported to act as a strong hydrophobic residue more than disulfide-bonding cysteine [18], addition of cysteine to lid region may increase the hydrophobicity of the lid region that is essential for interfacial activation. Similarly, a random mutation introduced at a loop connected to the lid helix involving arginine to cysteine substitution was reported to provide stability by enhancing hydrophobicity of the structure [30]. Qian and co-worker (2018) reported that substitution of glycine to a more hydrophobic residue, alanine improved the hydrophobic interactions of lipoxygenase thermostability by promoting the α-helix formation [31]. The indirect impact of substitution can be seen through other forces such as non-covalent interactions. Single substitution of an amino acid could lead to additional interactions due to the deviations in the degree of phi (φ) and psi (ψ) torsion angles, which could change the rotational freedom of peptide bonds. The side chain attached to the C_α_ of cysteine is more complex compared to glycine. Thus, this bulky side chain might alter the quality of rotation, which then affected the orientation of the neighboring residues. 

The subtle changes to the interior non-covalent interactions by the increase number of hydrogen bonds, salt bridges and cation-π interactions could possibly direct the improvement of conformational stability while maintaining the catalytic performance. Considering only one cysteine residue present in the structure, it is unlikely to engage in a disulfide bond hence we focused on the former interactions. Meanwhile, intramolecular cation-π interaction is generally known to associate with protein thermostability in which Lys and Tyr are commonly reported to favor the cation-π interaction in thermophilic proteins [32]. In our case, the newly evolved mutant demonstrated a stronger cation-π interaction at Lys 193 and Tyr265, which are located at the lid helix and β-strand, respectively. The strong cation-π interaction at lid 1 (residue 181–198) is found substantial to stabilize the helix structure at an elevated temperature hence accounts for its improved stability at 50 °C.

Improvement of thermostability in mutant G210C is also accompanied by additional hydrogen bonds formed throughout the structure. Consistent with our previous CD spectroscopy analysis, as these non-covalent interactions influenced the temperature-induced denaturation, a 10 °C higher in T_m_ was evidenced for mutant G210C [12]. Introduction of more hydrogen bonds could have increased the interior packing density and further led to increased Van der Waals interaction. In agreement, Vogt et al. (1997) [33] reported that the hydrogen bond was responsible for the thermostability of 16 families of proteins. We also identified an intraresidue hydrogen bond formed in Q208, close to the mutation point but was missing in the wild-type. Local side chain-main chain interactions have been recently suggested to cumulatively influence different parts of secondary structure and some may impart stability to irregular loops and turn [34]. Evidently, three salt bridge networks were observed in mutant G210C. The presence of additional salt bridge network may in part due to changes in the orientation of side chains of the pairs of oppositely charged residues that affect the geometric conformation. The bridging of K361 to D346 and R362 to E366 or D346 could stabilize the flexible Ca^2+^ binding site to increase rigidity. As protein unfolding is prone to regions with high thermal fluctuation, adding a salt bridge could minimize the fluctuation. Moreover, previous study indicated that increased of packing density on the protein surface could influence the resistance to high temperature [35], which is consistent with our finding on the exposed salt bridges observed. The presence of large number of charges and salt bridges in mutant G210C hence provide electrostatic strength to the structure to remain in its native folded state. A larger salt bridge network close to the active site which in turn increases the active site rigidity is significantly important to maintain its functionality. 

While the Ca^2+^ binding site remained in position near the catalytic site and widely reported to be important for catalysis, thermostability and preserving the active site conformation, the Zn^2+^ binding site could play its part primarily in maintaining the structural stability. As the Zn^2+^ interacting with three helices include α2, α3 and α5 and the loops near to the lids of both lipases, it is apparent that the metal binding site is responsible for keeping together all different parts of the protein. Similar observation has been reported in *S. hyicus* lipase where the tetrahedral coordination holds the secondary structure elements helix α3, loop α3-b2, helix α2, and loop α9-α10 and envisaged to maintain the structural stability [23]. A mutational study conducted by Biundo and co-workers (2017) [36] on the Zn^2+^ binding domain of esterase (which is also held by two aspartates and two histidines) has resulted in a drastic loss of thermostability when the ligands were substituted to leucine and phenylalanine, respectively. Furthermore, Cys210 is located in close association to the Zn^2+^ binding domain. Although sulfur in thiol group of cysteine residue has been occasionally reported to act as a ligand to metal, in our case, the thiol group was not part of the ligand binding residues. However, its presence in close contact with the lid may facilitate the maintenance of lid stability.

As previously reported, rT-M386 is exceptionally active at low temperatures and cold-adaptation is often correlated to conformational flexibility. However, proteins with greater flexibility and localized structural modifications are more susceptible to unfolding. When unfolding arises, the protein becomes extremely solvated, less compact and more flexible [37]. The dynamics of lipase at different temperatures can be understood by MD simulations. At 50 °C, mutant G210C maintained a steady RMSD values whereas rT-M386 conferred greater movement of the overall conformation. The flexibility around the altered site decreased suggesting the presence of a more rigid lid region. It was reported that, protein molecules necessitate both flexibility and rigidity to function, but improved compactness and high rigidity are needed to compensate the increased thermal fluctuations [37]. The improved compactness of G210C structure as denoted by its low Rg value may in part due to stabilization of secondary structure elements and loops by intramolecular interactions. In addition, most of the rigid thermostable proteins incline to endure in the native folded state at high temperature [21]. Solvent accessible surface area (SASA) is the surface area of a lipase that is accessible to a solvent. SASA is used to calculate the transfer of free energy from aqueous or polar solvent to a non-polar solvent as occur in lipid environment [38]. It was reported that, thermostable enzyme generally displays lower accessible area for high temperature adaptation [39]. A better folding process is typically accompanied by a significant decrease in SASA value and accessible surface areas of atoms are associated with their hydrophobicity [40]. Hence, replacement of glycine to cysteine attenuated the interaction with the solvents on the protein surface. A study suggested that when water molecules enter the structure, the hydrophobic forces will be destroyed. Consequently, this will expand the solvent-accessible surface area on the hydrophobic region and due to the pressure, the proteins begin to unfold [41]. Therefore, a strong hydrophobic interaction at protein surface is needed in order to protect the catalytic conformation by the intrusion of massive volume of solvent.

## 4. Materials and Methods 

### 4.1. Protein Sequence

The protein sequence was retrieved from NCBI GenBank (Accession No. EU814893, Protein ID: ACF28538). In previous studies, the mature protein (390 amino acids) was cloned in *E. coli* system and further work was conducted to generate a more stable lipase by deleting four C-terminal end residues [42,43]. The truncated AT2 lipase (designated as rT-M386) was randomly mutated generating a mutant with gly-cys mutation at position 210 (designated as G210C) [12]. Both rT-M386 and G210C lipase sequences were used in this study to predict the lipase models. 

### 4.2. Structure Prediction of rT-M386 and G210C Lipases

Structure prediction was performed using Yet Another Scientific Artificial Reality Application (YASARA version 10.4.11) software (Vienna, Austria) [44]. Template search was conducted using PSI-BLAST search at National Center for Biotechnology Information (NCBI) based on PDB database (http://www.ncbi.nlm.nih.gov/BLAST). In agreement with SWISS-MODEL template search (https://swissmodel.expasy.org/interactive), the crystal structure of *S. hyicus* lipase (PDB ID: 2HIH) shared the highest sequence identity (51%) and thus used for modeling. Simple energy minimization was performed for 300 steps by AMBER03 force field to find the lowest energy conformation, correct the atom position and covalent geometry. The final models were evaluated using PROCHECK (http://www.ebi.ac.uk/thornton-srv/software/PROCHECK/) and ERRAT2 [45]. Visualization of the structures was performed using YASARA and Chimera (version 1.13.1).

### 4.3. Covalent and Noncovalent Interactions Analysis

The total number and energy of hydrogen bonds, cation-π interactions and disulfide bond of rT-M386 and mutant G210C were calculated using YASARA while the salt bridge interaction analysis was performed using Evaluating the Salt BRIdges in Proteins (ESBRI) software (URL: http://bioinformatica.isa.cnr.it/ESBRI/) [46] 

### 4.4. Solvent Accessibility Prediction

To predict the surface accessibility and secondary structure of amino acids in both rT-M386 and mutant G210C, we used NetSufP2.0 (http://www.cbs.dtu.dk/services/NetSurfP/) [22] and input the primary amino acid sequences in FASTA format. 

### 4.5. Molecular Dynamics Simulations of rT-M386 and G210C Lipases

Simulations of rT-M386 and G210C lipase structures were performed using YASARA and the setup was done based on YASARA modeling and MD simulation program [47]. The periodic box was filled with explicit water molecules to a density of 0.997 g/mL. The counter ion concentration (Na^+^ and Cl^−^) was 0.9% and all ionizable protein groups were protonated according to their tabulated pKa values at pH 7 of the medium. Simulations were conducted at two temperatures 298 K (25 °C) and 323 K (50 °C) for a total of 20 ns at constant pressure. AMBER03 force field was used with a cut off 7.86 Å while Particle Mesh Ewald algorithm was used to compute long range electrostatic interactions. 3D coordinate snapshots were saved every 250 ps for a total of 20 ns simulation. Kinetic energy was initialized on each replica assigning random velocity vectors to all atoms (using Maxwell_Boltzman distribution). YASARA analysis toolkit was used to analyze MD trajectories by means of Root Mean Square Deviation (RMSD), Root Mean Square Fluctuation (RMSF), solvent accessible surface area (SASA) and radius of gyration (Rg). The simulation snapshots were visualized using YASARA.

## 5. Conclusions

The plasticity of rT-M386 as a cold-adapted lipase was clearly observed at both temperatures. While flexibility at low temperature is beneficial for low temperature catalysis, at elevated temperatures, the improved flexibility can alter the overall structure due to the unfolding of α-helices or β strands. As enzymatic activity needs an exact balance between stability and flexibility, rigidification of the global structure could be advantageous to achieve better stability at a high temperature. Random introduction of cysteine at the loop connecting the lid region has distinctively influenced the functional temperature. It is interesting that the increase in thermostability derived from one single mutation consequently leads to stabilization of secondary structure elements including the lid region by intramolecular non-covalent interactions and hydrophobic effect. The local effects could have altered the interaction between neighboring residues, hence indirectly affect the global conformation. More importantly, the gain of thermostability did not compromise its activity at low temperatures making it more catalytically efficient at a broad range of temperatures.

## Figures and Tables

**Table 1:** Comparison between the total number of hydrogen bonds and energy of rT-M386 and G210C lipases.

Lipases	No. of Hydrogen Bonds	Total Hydrogen Bond Energy [kJ/mol]
rT-M386	334	6848.55
G210C	349	7084.35

## Supplementary Materials

The following are available online, Figure S1: (a) PSI-BLAST result for rT-M386 and G210C lipase and (b) multiple sequence alignment of target and template, 2HIH; Figure S2: Ramachandran plot of (a) rT-M386 (b) G210C predicted models; Figure S3: Calcium and Zinc binding sites of rT-M386 and G210C; Figure S4: NetSurP results (a) rT-M386 (b) G210C.
