# Supplementary material for: Insight into Improved Thermostability of Cold-Adapted Staphylococcal Lipase by Glycine to Cysteine Mutation
Source: Molecules. 2019 Aug 30;24(17):3169. doi: 10.3390/molecules24173169 (PMC6749283; doi:10.3390/molecules24173169)

## Supporting Information

**Figure S1. a) PSI-BLAST result for rT-M386 and G210C lipase and b) multiple sequence alignment of target and template, 2HIH.** (https://blast.ncbi.nlm.nih.gov)

**a)**

| Description | Max score | Total score | Query cover | E value | Ident | Accession |
|---|---|---|---|---|---|---|
| Chain A, Crystal Structure Of Staphylococcus Hyicus Lipase | 390 | 390 | 98% | 5e-134 | 51% | 2HIH_A |
| Chain A, Crystal Structure Of Lipase From Geobacillus Stearothermophilus T6 Methanol Stable Variant H86y/a269t | 228 | 228 | 94% | 3e-71 | 36% | 4X7B_A |
| Chain A, Crystal Structure Of Lipase From Geobacillus Stearothermophilus T6 Methanol Stable Variant H86y/a269t/r374w | 228 | 228 | 94% | 5e-71 | 36% | 4X85_A |
| Chain A, Structure Of Lipase 1 From Pelosinus Fermentans | 219 | 219 | 94% | 6e-68 | 39% | 5AH0_A |
| Chain A, Structure Of Esta From Clostridium Botulinum | 218 | 218 | 89% | 2e-66 | 38% | 5AH1_A |
| Chain A, Crystal Structure Of A Lipase From Geobacillus Sp. Sbs-4s | 206 | 206 | 95% | 2e-62 | 35% | 3AUK_A |
| Chain A, Crystal Structure Of T1 Lipase | 205 | 205 | 94% | 3e-62 | 35% | 2DSN_A |
| Chain A, An Organic Solvent Tolerant Lipase 42 | 205 | 205 | 94% | 3e-62 | 35% | 4FKB_A |
| Chain A, Crystallization And 3d Structure Elucidation Of Thermostable L2 Lipase From Thermophilic Locally Isolated Bacillus Sp. L2 | 205 | 205 | 94% | 3e-62 | 35% | 4FDM_A |
| Chain A, Crystal Structure Of D311e Lipase | 204 | 204 | 94% | 4e-62 | 35% | 3UMJ_A |

**b)**

| Score | Expect | Method | Identities | Positives | Gaps |
|---|---|---|---|---|---|
| 396 bits(927) | 7e-113 | Compositional matrix adjust. | 196/383(51%) | 223/383(58%) | 7/383(1%) |

```
Query  6     KNQYPVVFVHGFVGLVGEDSFSMYPNYWGGTKYNVKQELTKLGYRVHEANVGAFSSNYDR  65
             KN  P VFVHGF G VGE       NYWGGTK N    L K GY   EA V A  SN +R
Sbjct  50    KNKDPFVFVHGFTGFVGEVA-AKGENYWGGTKANLRNHLRKAGYETYEASVSALASNHER  108

Query  66    AVELYYYIKGGRVDYGAAHAAKYGHKRYGRTYEGIMPDWEPGKKIHLVGHSMGGQTIRLM  125
             AVELYYY KGGRVDYGAAH  KYGH RYG TYEG++ DW PG  +H +GHSMGGQTIRL+
Sbjct  109   AVELYYYLKGGRVDYGAAHSEKYGHERYGKTYEGVLKDWKPGHPVHFIGHSMGGQTIRLL  168

Query  126   EHFLRNGNQEEIDYQRQYGGTVSDLFKGGQDNMVSTITTLGTPHNGTPAADKLGSTKFIK  185
             EH+LR G+  EI YQ Q GG +S+LFKGGQDNMV  ITT  TPHNGT A D  G T  I
Sbjct  169   EHYLRFGDKAEIAYQQQHGGIISELFKGGQDNMVTSITTIATPHNGTHASDDIGNTPTIR  228

Query  186   DTINRIGKIGGTKALDLELGFSQWGFKQKPNESYAEYAKRIANSKVWETEDQAVNDLTTA  245
             +                + G  +WGFK K  ES  +Y KRIA SK+W+ ED    DLT
Sbjct  229   NILYSFAQMSSHLG-TIDFGMDHWGFKRKDGESLTDYNKRIAESKIWDSEDTGLYDLTRE  287

Query  246   GAEKLNQMTTLNPNIVYTSYTGAATHTGPLGNEVPNI-RQFPLFDLTSRAIGGDDNKNVR  304
             GAEK NQ T LNPNI Y  YTG ATH   LG  + +   +F    LT   IG  D+   R
Sbjct  288   GAEKINQKTELNPNIYYKTYTGVATHETQLGKHIADLGMEFTKI-LTGNYIGSVDDILWR  346

Query  305   VNDGIVPVSSSLHPSDEAFKKVGM-MNLATDKGIWQVRPVQYDWDHLDLVGLDTTDYKRT  363
              NDG V   SS HPSDE  K +    N    KG WQV P    WDH D +G D  D K
Sbjct  347   PNDGLVSEISSQHPSDE--KNISVDENSELHKGTWQVMPTMKGWDHSDFIGNDALDTKHS  404

Query  364   GEELGQFYKSMINNMLKVEELDG  386
               EL  FY S  + ++ +E  +
Sbjct  405   AIELTNFYHSISDYLMRIEKAES  427
```

**Figure S2. Ramachandran plot of** a) rT-M386 b) G210C predicted models. Color are represented as follows; most favored region (red), additional allowed region (brown), generously allowed region (yellow) and disallowed region (white).

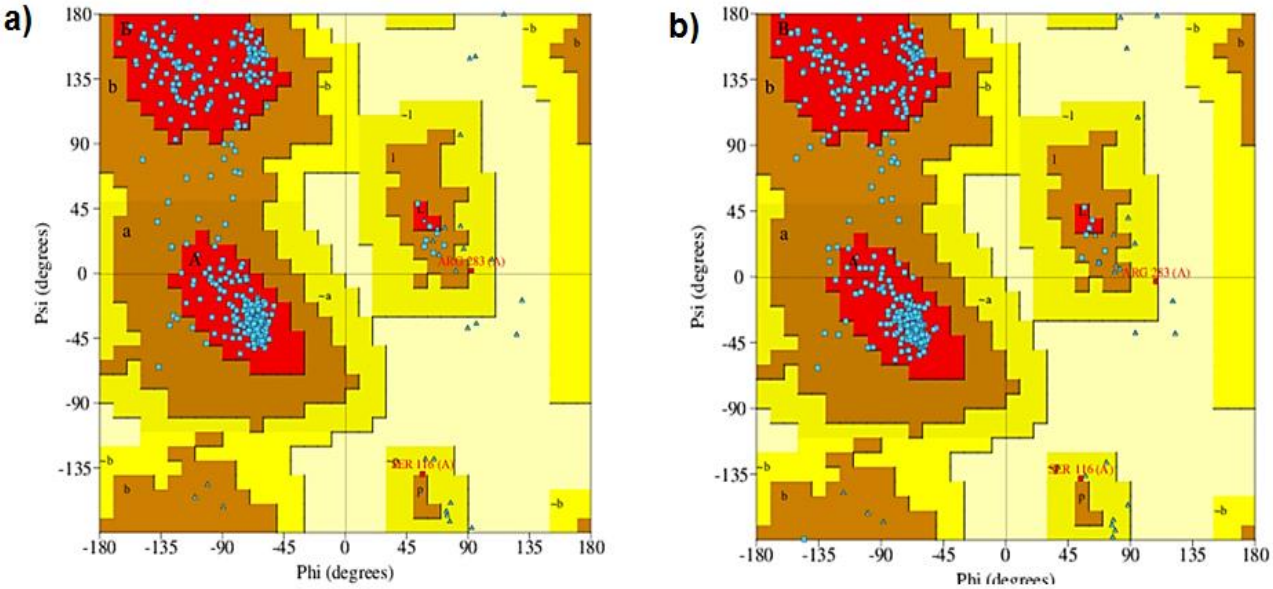


**a)**

1. Ramachandran Plot statistics

| | | No. of residues | %-tage |
|---|---|---|---|
| Most favoured regions | [A,B,L] | 293 | 89.3%* |
| Additional allowed regions | [a,b,l,p] | 33 | 10.1% |
| Generously allowed regions | [~a,~b,~l,~p] | 2 | 0.6% |
| Disallowed regions | [XX] | 0 | 0.0% |
| Non-glycine and non-proline residues | | 328 | 100.0% |
| End-residues (excl. Gly and Pro) | | 1 | |
| Glycine residues | | 43 | |
| Proline residues | | 14 | |
| Total number of residues | | 386 | |

**b)**

1. Ramachandran Plot statistics

| | | No. of residues | %-tage |
|---|---|---|---|
| Most favoured regions | [A,B,L] | 298 | 90.6% |
| Additional allowed regions | [a,b,l,p] | 29 | 8.8% |
| Generously allowed regions | [~a,~b,~l,~p] | 2 | 0.6% |
| Disallowed regions | [XX] | 0 | 0.0% |
| Non-glycine and non-proline residues | | 329 | 100.0% |
| End-residues (excl. Gly and Pro) | | 1 | |
| Glycine residues | | 42 | |
| Proline residues | | 14 | |
| Total number of residues | | 386 | |

**Figure S3. Calcium binding site of** a) rT-M386 b) G210C and **Zinc binding site of** c) rT-M386 d) G210C. The distances (Å) between each interaction are as indicated in the diagram. Figures are generated using the PDBsum (http://www.ebi.ac.uk/pdbsum).

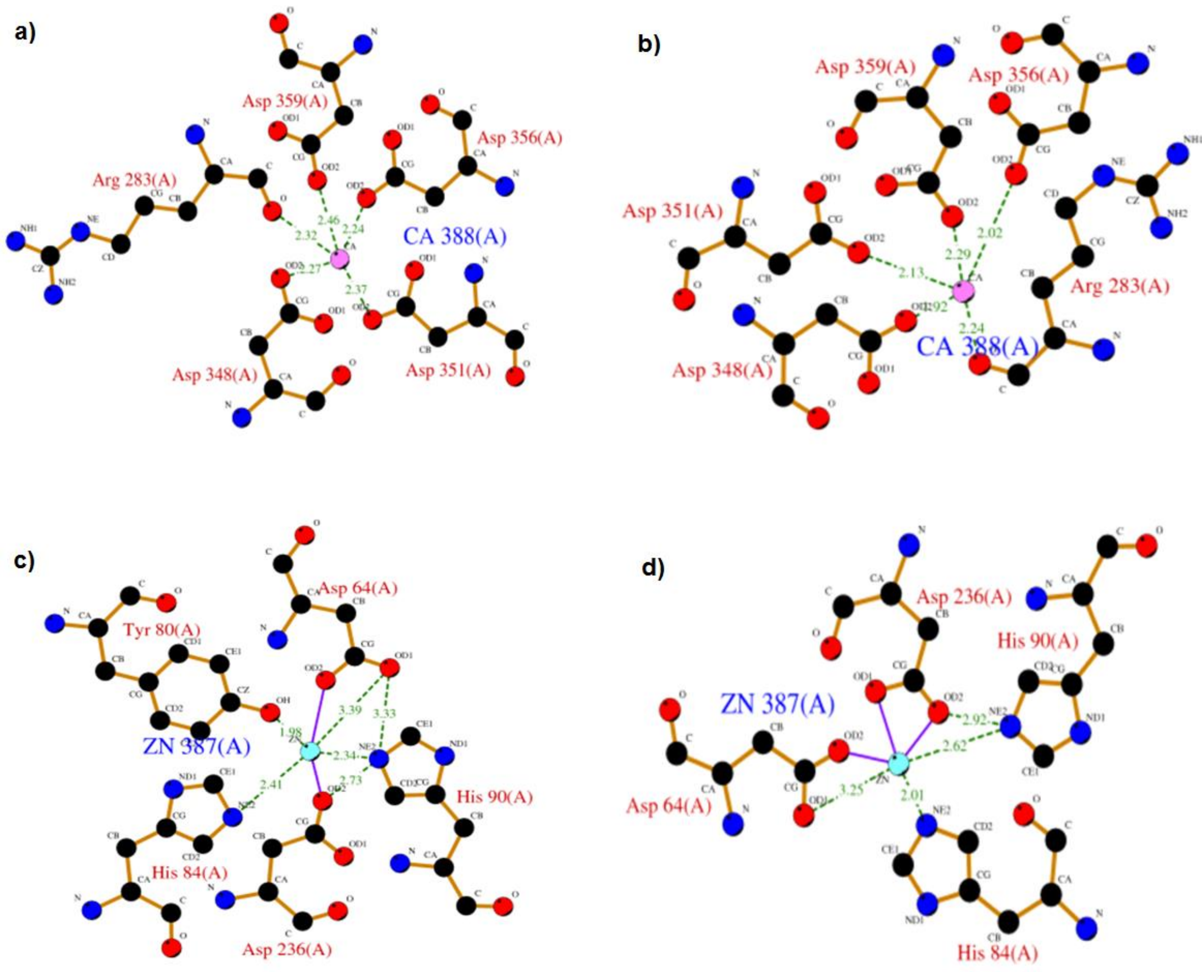

**Figure S4. NetSurP results for** a) rT-M386 and b) G210C. Residue 210 is highlighted in grey and the RSA value is shown in the box. Results are generated using NetSurP-2.0 (http://www.cbs.dtu.dk/services/NetSurfP/)

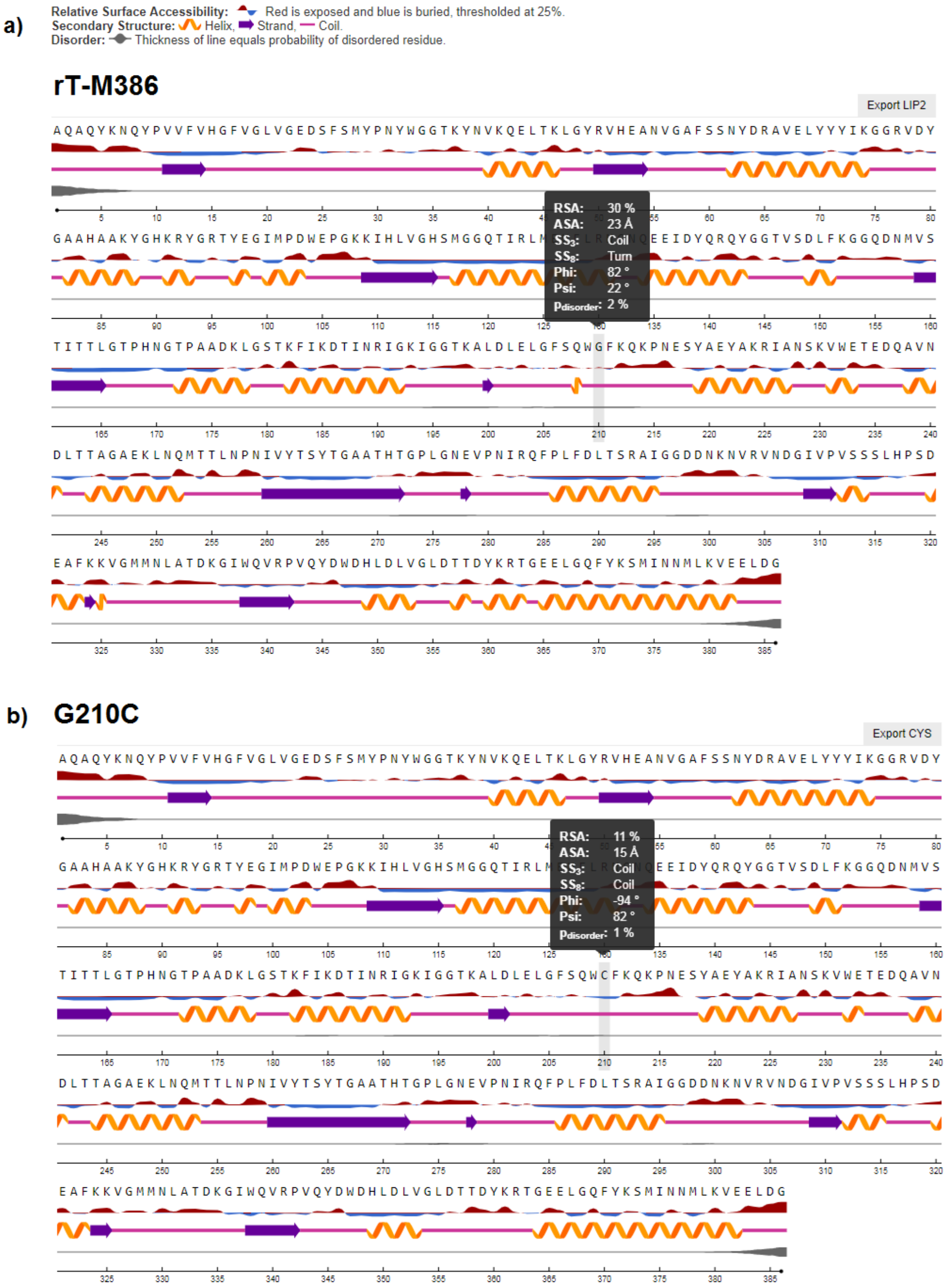

Supplement: Supplementary file 1 [file molecules-24-03169-s001.pdf]
